# A Promising Insight: The Potential Influence and Therapeutic Value of the Gut Microbiota in GI GVHD

**DOI:** 10.1155/2022/2124627

**Published:** 2022-05-05

**Authors:** Jiahua Li, Xueyan Zhang, Yiru Chen, Qingqing Zheng, Mingyi Zhao, Hua Jiang

**Affiliations:** ^1^Department of Hematology, Guangzhou Women and Children's Medical Center, Guangzhou Medical University, Guangzhou, Guangdong 510623, China; ^2^Department of Pediatrics, The Third Xiangya Hospital, Central South University, Changsha, Hunan 410013, China; ^3^Xiangya School of Medicine, Central South University, Changsha, Hunan, China

## Abstract

Allogeneic hematopoietic cell transplantation (allo-HSCT) is a reconstruction process of hematopoietic and immune functions that can be curative in patients with hematologic malignancies, but it carries risks of graft-versus-host disease (GVHD), thrombotic microangiopathy (TMA), Epstein–Barr virus (EBV) infection, cytomegalovirus infection, secondary hemophagocytic lymphohistiocytosis (sHLH), macrophage activation syndrome (MAS), bronchiolitis obliterans, and posterior reversible encephalopathy syndrome (PRES). Gastrointestinal graft-versus-host disease (GI GVHD), a common complication of allo-HSCT, is one of the leading causes of transplant-related death because of its high treatment difficulty, which is affected by preimplantation, antibiotic use, dietary changes, and intestinal inflammation. At present, human trials and animal studies have proven that a decrease in intestinal bacterial diversity is associated with the occurrence of GI GVHD. Metabolites produced by intestinal bacteria, such as lipopolysaccharides, short-chain fatty acids, and secondary bile acids, can affect the development of GVHD through direct or indirect interactions with immune cells. The targeted damage of GVHD on intestinal stem cells (ISCs) and Paneth cells results in intestinal dysbiosis or dysbacteriosis. Based on the effect of microbiota metabolites on the gastrointestinal tract, the clinical treatment of GI GVHD can be further optimized. In this review, we describe the mechanisms of GI GVHD and the damage it causes to intestinal cells and we summarize recent studies on the relationship between intestinal microbiota and GVHD in the gastrointestinal tract, highlighting the role of intestinal microbiota metabolites in GI GVHD. We hope to elucidate strategies for immunomodulatory combined microbiota targeting in the clinical treatment of GI GVHD.

## 1. Introduction

Allogeneic hematopoietic stem cell transplantation (allo-HSCT) is considered to be an effective treatment for various hematological malignancies, bone marrow failure diseases, genetic metabolic diseases, and immunodeficiency diseases [1–3]. Hematopoietic stem cells are extracted from the bone marrow or peripheral blood of allogeneic donors and infused into recipients who have received myeloablative conditioning regimens. In addition to hematopoietic stem cells, the transplant also contains allogeneic donor T cells, which may attack the host tissue cells and lead to the occurrence of graft versus host disease (GVHD). Acute GVHD (aGVHD) is an immune-mediated process that occurs after allo-HSCT characterized by the response of donor antigen-specific lymphocytes (mainly T cells) to host allogeneic antigens and inflammatory cytokine cascades, and its progress can be briefly summarized in three consecutive stages, namely, the activation of antigen-presenting cells; the activation, proliferation, differentiation, and migration of donor T cells, as well as inflammatory cytokines; and the cell-mediated destruction of target tissue [4].

Affecting 20% to 80% of allo-HSCT recipients during the use of long-term immunosuppressants for prophylaxis, aGVHD is still the major complication after allo-HSCT, and it can affect the gut, liver, lungs, and skin. Primarily due to the increased use of unrelated and/or HLA-mismatched donors and granulocyte-colony-stimulating factor, an increasing incidence of GVHD has been observed in recent years [5]. Severe GI GVHD, often associated with poor patient outcomes, is one of the leading causes of transplant-related death. GI GVHD breaks down the mucosal immune system by attacking the intestinal crypt and its stem cell niche, and the risk factors for mortality include corticosteroid resistance, age > 18 years, increased serum bilirubin, and overt gastrointestinal bleeding [6, 7].

The human body is colonized by a large number of microorganisms, most of which are bacteria, but also including viruses, fungi, and archaea. These microorganisms are usually called the intestinal microbiota, and their related community is called the intestinal microbiome [8]. There are relative differences in the composition of microorganisms between different sites in the same individual, but they all change dynamically. The intestine is the main site for microbial colonization, with approximately 100-150 bacterial species forming 100 trillion cells. The structural characteristics of a healthy bacterial community are conducive to the development and maturity of the host immune system, digestion of food, synthesis of essential amino acids, short-chain fatty acids and vitamins, regulation of the immune response, and enhancement of the resistance to pathogen infection [9–12].

Gut bacteria are in equilibrium with the host innate immune system and help to maintain homeostasis, which when altered directly impacts host health. Recent studies have found that certain intestinal bacteria are associated with the outcomes of allo-HSCT transplantation, and disruption of the normal structure of the gut microbiota is related to the risk of GVHD [10, 13]. Taur et al. examined the effect of intestinal diversity on posttransplant mortality. Fecal specimens collected from 80 recipients were divided into low-, medium-, and high-diversity groups, with 3-year overall survival rates of 36%, 60%, and 67%, respectively. The transplant-related mortality (TRM) was 53%, 23%, and 9%, suggesting that intestinal flora may be a pivotal factor in the success or failure of allo-HSCT [14]. In a multicenter study, 8767 fecal samples obtained from 1362 patients undergoing allo-HSCT were characterized by a loss of diversity and domination by single taxa, identifying an association between a high diversity of intestinal microbiota at the time of neutrophil engraftment and low mortality [15]. Ingham et al. revealed that the diversity of the intestinal microbiota decreased within one month after transplantation, and the lowest alpha diversity (inverse Simpson) was observed from the day of HSCT to week +3 for the gut [16].

In this article, the role and changes in the intestinal flora during the occurrence and development of GVHD are introduced, which provides a theoretical basis for the prevention and treatment of intestinal GVHD and summarizes the most current treatment strategies.

## 2. Intestinal Flora, Intestinal Mucosa, and GVHD

### 2.1. The Structure and Physiological Function of the Intestinal Epithelium

In general, the intestinal epithelium consists of a single layer of tightly connected cells that effectively insulate the direct exchange of intestinal contents, which is called the “intestinal barrier” [17]. The presence of tight junctions in the intestinal epithelium effectively prevents microorganisms, intestinal contents, and antigen molecules from entering the body from the gastrointestinal lumen but allows digestive products and water to enter the body [18]. This is the physical intestinal barrier. In addition, the gut also has a mucus-antimicrobial peptide barrier that regulates epithelial permeability and an immune barrier comprised of the body's innate and specific immune systems [19, 20]. These three barriers work together to maintain homeostasis in the intestinal environment. If the intestinal barrier is damaged due to various reasons, the basic intestinal functions cannot be carried out and the growth environment, distribution, and abundance of the intestinal flora changes [21]. This will interfere with many normal physiological processes of the body, such as immunity and metabolism, resulting in undesirable consequences [22].

The human gastrointestinal system is the largest digestive and endocrine organ in the human body and has a close relationship with other systems in the body. A large number of cells distributed in the intestinal epithelium play important roles in regulating absorption, the secretion of substances, immune activation, and flora ecology. These include enterocytes, goblet cells, Paneth cells, tuft cells, enteroendocrine cells, and M cells. Generally, goblet cells secrete mucus to form a protective barrier. Paneth cells secrete antimicrobial peptides and growth factors that stimulate the production of epithelial stem cells [23]. Recent studies have shown that congenital lymphoid cells type 3 (ILC3) can be activated by cytokines produced by mononuclear phagocytes IL-23, IL-1*β*, and TL1A [24], and they support the production of antimicrobial peptides and mucins by intestinal epithelial cells (IECs) through the secretion of IL-22 [24], ensuring the spatial isolation of microorganisms from intestinal tissue [25, 26]. This effect also preserves signal transduction between the intestinal microbiota and IECs, maintaining intestinal immune and metabolic homeostasis [27].

### 2.2. Intestinal Flora and Immunity

The intestinal flora remains a heated topic in recent years because it seems to have both subtle and obvious influences on the physiological and pathological state of various organs and systems of the body. Microbes, such as bifidobacteria, Lactobacillus, *Escherichia coli*, Enterococcus, *Clostridium perfringens*, and Pseudomonas, have been proven to colonize our guts [28, 29]. According to previous studies, intestinal flora can not only directly promote the development of lymphocytes, such as B cells in intestinal-associated lymphoid tissue (GALT) [30], but also stimulate the expression of PRRs on intestinal mucosal epithelial cells or immune cells and induce the maturation of intestinal mucosal lymphoid tissue through pathogenesis-associated molecular patterns (PAMPs) [31, 32].

The balance of intestinal flora has been linked to the prevention of immune-mediated diseases such as inflammatory bowel disease (IBD) and asthma. One study found an increased incidence of IBD and allergic asthma in germ-free (GF) mice relative to pathogen-free mice [33]. This is due to increased expression of the chemokine ligand CXCL16 in the gut and lungs in the absence of intestinal flora [34]. Immobile natural killer T (iNKT) cells accumulate in the lamina of the colon and lung, and the mice become less resistant to environmental exposures. Coombes et al. also demonstrated that the intestinal microbiota could promote the expansion of CD4+ T cells produced by IFN-*γ* in colitis, thereby exacerbating the occurrence of intestinal inflammation [35].

New research has shifted the focus from the effects of gut microbiota on immune cells to proteins and genes, seeking to understand the nature of the effects of gut microbiota on the immune system. The Gimap5 gene is one of the regulators of hematopoietic integrity and lymphocyte homeostasis. Barnes et al. verified that in Gimap5-deficient mice, T-cell loss and B-cell immobility aggravated the occurrence of microbiome-dependent wasting disease and intestinal inflammation [36]. In IECs, *C. rodentium* promoted the differentiation of Th17 cells in the colonic lamina propria by upregulating Nos2, Duoxa2, and Duox2 [37]. However, the impact of the gut flora on genes still requires much research and data to tease out the connections.

### 2.3. Intestinal Flora and ROS

According to Saxena et al., reactive oxygen species (ROS) are involved in the function of intestinal stem cells (ISCs) and the IEC defense against foreign bacteria [38]. Saxena et al. conducted research on mice and found that loss of the autophagic protein 5 (ATG5) gene resulted in impaired ISC-dependent intestinal recovery. The use of ROS induction reagents can significantly reduce the number and regeneration of ISCs [39]. Treating G5 knockout mice with antioxidants could thus rescue the defect [40]. Disruption of the intestinal flora activates intestinal epithelial cells. In nod2-deficient or ATG16L1 mice, bacteria enter their intestinal epithelial cells and increase IL-8 production [39]. In other words, intracellular mitochondrial oxidative stress may change the bacterial survival rate and promote the occurrence and development of intestinal inflammatory diseases such as IBD.

In addition, Larabi et al. mentioned that the intestinal flora may also affect ROS production during the regulation of autophagy [41]. Yue et al. reported that trimethylamine N-oxide prime NLRP3 produced by intestinal flora could be involved in the development of IBD by inhibiting ATG16L1, SQSTM1, and LC3-II and increasing ROS production in a dose- and time-dependent manner [42]. Autophagy regulation of mitochondrial ROS plays a key role in the regulation of macrophage migration inhibitory factor (MIF) secretion. In the presence of bacterial LPS, drug inhibition or siRNA silencing of Atg5 can enhance MIF secretion by monocytes and macrophages in a mitochondrial ROS-dependent manner [43].

### 2.4. Intestinal Flora and Infection

If the permeability of intestinal epithelial cells changes or the tight connections between cells are lost, the intestinal flora can enter the body through the intestinal wall. This can lead to ectopic intestinal flora and infection. In addition, abnormal secretion of antibiotics, abnormal immunity, changes in phage activity, and abnormal colonization of exogenous bacteria in the intestinal tract may change the growth environment and disrupt the balanced state of the normal intestinal flora, thus leading to infection [44, 45]. Colicin FY is a bacteria-produced antibacterial agent that has a specific growth inhibition effect on *Yersinia enterocolitica*, the pathogen of gastrointestinal yersinia disease. *E. coli*-producing colicin FY inhibited the growth of pathogenic *Yersinia enterocolitica*. When mice were treated with streptomycin, the *E. coli* strain producing colicin-FY inhibited the progression of enterocolitis infection [46]. These results indicate that colicin FY has in vivo antibacterial activity and can be used for the treatment of enterocolitis infection.

The human intestinal flora can play a certain immune activation and antibacterial role. However, overuse of antibiotics gives rise to the abundance of the gut bacteria itself being disrupted, making infections more easily invading [44, 47]. Becattini et al. demonstrated that infection with *Listeria monocytogenes* in immunodeficient or chemotherapy mice could cause sepsis and meningitis. Taking antibiotics can worsen these infections. However, if the symbiotic flora with in vitro antibacterial activity is transplanted into germ-free mice, it will induce the mice to produce antibodies and start the immune response, thus playing a defensive role against infection [45]. Research by Corr et al. also found that *Lactobacillus salivarius* UCC118 produces bacteriocin Abp118 in vivo, which significantly protects mice from invasive *Listeria monocytogenes* infection. A nonproducing mutant of *Lb. salivarius* did not produce bacteriocin Abp118, nor did it protect mice from two strains of *Listeria monocytogenes* [48]. This finding indicates that the intestinal flora induces bacteriocin production as an important protective agent.

### 2.5. GVHD Causes Damage to the Intestinal Mucosa

Recent studies have demonstrated that ISCs are a target of GVHD. With the development of GVHD, ISC is impaired. Takashima et al. found that injection of the Wnt agonist R-spondin1 (R-SPO1) prevented ISC injury, enhancing the repair of damaged intestinal epithelial cells [49]. It inhibits the subsequent inflammatory cytokine cascade reactions. IL-22 is an important regulator of tissue sensitivity to GVHD and a protective factor against ISC in inflammatory bowel injury [23]. Hanash et al. showed that intestinal IL-22 is increased under pretransplant regulation and is produced by IL-23-responsive innate lymphoid cells (ILCs) after bone marrow transplantation [50]. GVHD, however, reduced the ILC frequency and IL-22 abundance, leading to increased crypt cell apoptosis [50]. ISC depletion results in the loss of intestinal epithelial integrity.

L cells proved to be a target of GVHD. Acute GVHD reduced glucagon-like peptide-2 (GLP-2) levels produced by intestinal L-cells in mice and in patients with graft-versus-host disease [51]. GLP-2 can promote the regeneration of ISCs and the production of antimicrobial peptides, reduce the expression of apoptosis-related genes, and cause changes in the intestinal microbial community [52, 53]. Norona et al. demonstrated that in a mouse model, treatment with a GLP-2 agonist alleviated emergent acute GVHD [54].

Goblet cells are also reduced in GVHD. Goblet cell loss as shown on patient biopsy is associated with severe GI GVHD and a poor prognosis [55, 56]. Ara et al. showed that GVHD was aggravated in mice lacking the antibiotic Lypd8. IL-25-pretreated goblet cell preservation maintained the intestinal barrier and attenuated GVHD [57]. This demonstrates that the loss of goblet cells destroys the mucus layer in the colon and allows bacterial translocation.

## 3. The Interaction of Gut Microbiota and GI GVHD

AGVHD can occur in each segment of the digestive tract. If it occurs in the oropharynx, the main manifestations are oral pain, pain upon swallowing, loss of appetite, blisters, aphthosis, and gingivitis [58]. The probability of occurrence in the esophagus is low, predominantly manifested by nausea and vomiting [59]. If it occurs in the stomach and duodenum, latent symptoms may appear first, including loss of appetite, bloating, indigestion, and weight loss. The worsening symptoms can progress to nausea, persistent vomiting, upper abdominal pain and upper gastrointestinal tract bleeding, and melena. If it occurs in the small and large intestine, it mainly manifests as abdominal pain, diarrhea, and bloody stool. It is worth noting that the toxicity and opportunistic infections of chemotherapy drugs can also cause gastrointestinal symptoms similar to aGVHD before implantation. In addition, drug toxicity, aGVHD, and infection can coexist at the same time, which adds much difficulty to the clinical diagnosis of intestinal GVHD [60]. Studies have shown that intestinal bacteria are inextricably linked to the occurrence of intestinal GvHD and infectious complications after allo-HSCT. It has been found that allo-HSCT is associated with a significant reduction in intestinal microbial diversity and is considered to be a combined effect of multiple factors, such as pretreatment, antibiotic use, dietary changes, and intestinal inflammation [13, 61, 62], and these factors may also be related to the occurrence of intestinal GVHD.

Here, we focused on reviewing the mechanism of GI GVHD, changes in the gut microbiota after allo-HSCT, and the relationship between the gut microbiota and GVHD.

### 3.1. Related Mechanisms of aGVHD

In the pretransplantation stage, host tissue damage caused by radiotherapy and chemotherapy can mediate damage-related molecular patterns (DAMPs) (such as adenosine triphosphate (ATP) [63], high-mobility group protein B1 (HMGB1) [64], NLRP3 inflammasome, uric acid, [65] heparan sulfate [66], and the release of inflammatory cytokines (such as IL-6 [67] and TNF [68]). Pathogen-related molecular patterns (PAMPs) include bacterial degradation products (lipopolysaccharides, lipoproteins, peptidoglycans, and flagellin), fungal degradation products (such as *β*-glucan and a-mannan), and viral nucleic acids [69]. After these substances are released, they can enter the blood through the damaged gastrointestinal mucosa, activate the immune system, and trigger a cascade of inflammatory cytokines, resulting in increased expression of major histocompatibility complex (MHC) antigens and adhesion molecules, thereby improving the ability of donor T cells to recognize host allogeneic antigens [70]. In addition, DAMPs and PAMPs can activate antigen-presenting cells (APCs) derived from recipients and donors, such as dendritic cells [71] and macrophages [72].

Under the costimulatory signal interacting with host antigen-presenting cells after allo-HSCT, donor T cells expressing TCR are activated after recognizing the APCs of allogeneic antigens in HLA class I and class II. Current research shows that donor CD8 T cells are mainly activated by the recipient's hematopoietic APC, while donor CD4 T cells are activated by the recipient's gastrointestinal nonhematopoietic APC [73, 74]. After that, the activated donor T cells are expanded into the Th1, Th2, and Th17/Tc17 subtypes [75].

In the third stage of aGVHD, cytotoxic effects and cytokines directly and indirectly mediate tissue damage, respectively. Activated T cells migrate to the main GVHD target organs (intestine, liver, and skin), causing target tissue damage, whose histological manifestation is epithelial cell apoptosis. CD4+ Th cells, CTLs, and NK cells interact with Fas-FasL, TNF, or TNF-related apoptosis-inducing ligands or the release of perforin and granzyme stored in the cells, exerting cell-dependent cytotoxicity [76]. DAMPs and PAMPs stimulate recipient cells to secrete more cytokines (such as TNF, IL-1, IL-6, IL-33, IL-12, IL-23, type 1 IFN, and IFN-*γ*) and chemokines (such as CCL5 and CXCL2) to enhance the allogeneic antigen presentation of the receptor APC and the expression of costimulatory molecules and cytokines, thereby initiating an inflammatory cascade [77]. In addition, bacteria that penetrate the intestinal wall can activate neutrophils and recruit them to the site of bacterial infection. Neutrophils can directly cause tissue injury by releasing ROS, sequentially damaging the gastrointestinal tract [78]. CXCL2 plays a critical role in the recruitment of macrophages to target organs during the occurrence of GVHD; the higher the proportion of macrophages (M1/M2), the higher the incidence of grades II-IV aGVHD [79, 80].

### 3.2. How GVHD Damages the Intestinal Mucosa

GI GVHD targets small intestinal stem cells (ISCs), leading to epithelial cell apoptosis, and its most significant histology is apoptotic bodies in the crypt regeneration cavity. The histological severity of clinical GI GVHD is classified according to the degree of crypt damage: isolated apoptotic bodies without crypt loss (level 1), crypt apoptosis with individual crypt loss (level 2), crypt apoptosis with loss of two or more contiguous crypts (level 3), and extensive crypt loss and epithelial denudation (level 4) [81, 82]. However, the GI GVHD endoscopic manifestations are multifarious and can lack any characteristic changes, and different stages of progressive mucosal inflammation can be observed, including normal mucosa, mucosal edema and seepage, loss of blood vessels, erythematous lesions, mucosal erosions, superficial ulcers, white plaques, severe mucosal collapse, and mucosal peeling [60]. During the occurrence of GI GVHD, the destruction of the intestinal mucosal barrier mediated by immune disorders and intestinal dysfunction often manifests as severe diarrhea. Hence, daily diarrhea volume, intestinal obstruction, and bleeding symptoms are commonly used in clinical practice for grading [83].

**Figure 1:**
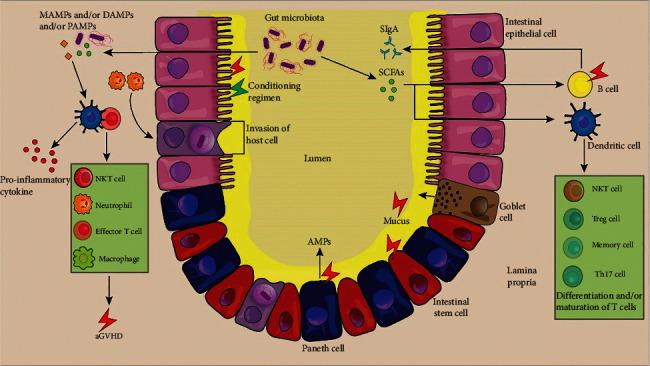
Intestinal barrier damage by GVHD.

During the pretreatment process of allo-HSCT, the balance of intestinal bacteria in the recipient is disrupted by total body radiotherapy or chemotherapy, resulting in impaired intestinal mucosal barrier function. Intestinal bacteria, MAMPs (microorganism-related molecular patterns), and PAMPs are translocated to the lamina propria, which are recognized by Toll-like receptors and presented to T cells by DCs, activating effector T cells, further aggravating the intestinal mucosal injury [84]. At the same time, the infiltration of neutrophils into the small intestine promotes the development of GI GVHD through the production of reactive oxygen species, resulting in increased tissue damage [85]. In addition, GVHD can also attack B cells, goblet cells, Paneth cells, and mucous layers at the bottom of the crypts and aggravate intestinal inflammation and bacterial translocation, greatly increasing the susceptibility of the recipient to infection [13, 84] (Figure 1). Studies have shown that Paneth cells [86] and the significant reduction in AMPs secreted by them are related to the severity of GVHD. Meanwhile, severe GI GVHD and Paneth cell counts are related to the loss of microbial diversity [87]. Paneth cells support Lgr5+ ISCs for normal epithelial cell regeneration through the epidermal growth factor (EGF), WNT3, and Notch ligand signaling pathways, while IL-22 produced by innate lymphoid cells (ILCs) after intestinal injury directly acts on ISCs, enhancing ISC-mediated epithelial cell regeneration [88].

In an ileal organoid model, an increased level of IL-22 limited the proliferation of ISCs but facilitated the proliferation of transit-amplifying TA progenitor cells [89]. Goblet cells and the mucin they secrete form a mucus barrier that prevents pathogens from invading the mucosa and causing intestinal inflammation (Figure 1). In a mouse model of post-allo-HSCT, GVHD targeting intestinal goblet cells destroys the double-layer structure of the colon mucus and induces bacterial migration. IL-25 can promote the differentiation and maturation of goblet cells in the large intestine. Prior to transplantation, IL-25, which depends on Lypd8 (an antibacterial molecule produced by intestinal cells in the colon that can inhibit the activity of flagella bacteria), can reduce IFN-*γ* and IL-6 in the plasma, protect goblet cells from GVHD, and prevent bacterial migration. Clinically, a low number of goblet cells in the colon and severe GI GVHD are related to a poor transplant outcome [57]. Moreover, goblet cells can also present antigens in the lumen to dendritic CD103+ cells to induce adaptive immune responses [90], indicating that goblet cells may be involved in GI GVHD before they become targets of attack.

### 3.3. The Relationship between Intestinal Bacteria and GI GVHD

The microbial communities that colonize healthy intestines comprise a large and diverse community of bacteria, such as Firmicutes, Bacteroidetes phyla, Proteobacteria, Actinobacteria, Verrucomicrobia, and Fusobacteria, that have coevolved with the host intestinal immune system. In a healthy gut, the obligate anaerobes Clostridium and Bacillus usually maintain the advantages of facultative anaerobic Enterobacteriaceae. Doki et al. showed a significantly higher abundance of the phylum Firmicutes and a lower tendency for Bacteroidetes in aGVHD patients than in nonaGVHD patients [91]. Diet plays a vital role in the composition of the gut microbiota, and dietary changes can lead to disturbances in the structure of the gut microbial community. In addition, different dietary habits may produce differences in the gut microbiota among healthy individuals [92–94]. During allo-HSCT, due to the influence of antibiotics, intestinal inflammation, and dietary changes, a significant decrease in the diversity of gut microbiota can be observed [62]. The decrease in the diversity of the gut microbiota, colonization with multidrug-resistant bacteria, and infections are associated with an increased risk of GI GVHD [95]. It is worth noting that colonization with multidrug-resistant bacteria also significantly increased nonrelapse mortality and systemic infections, resulting in a decrease in the overall survival rate after allo-HSCT [96].

Gut commensal microbiota and its metabolites, such as lipopolysaccharides, short-chain fatty acids, and secondary bile acids, affect local and systemic immunity through direct and indirect interactions with immune cells, thereby aggravating or alleviating GVHD [97]. During allo-HSCT, Enterococcus, Streptococcus, and Proteobacteria can dominate the intestinal bacteria with a significant decrease in diversity [14]. The increase in enterococci belonging to Lactobacillus spp. can damage the integrity of the intestinal epithelial cell barrier and stimulate macrophages to produce TNF [13], which may aggravate inflammation and damage the intestinal barrier. As the most important source of nutrition for enterococci, lactose can promote the expansion of enterococci after allo-HSCT. Patients with lactose intolerance find it difficult to digest lactose, so it can accumulate in their intestines, and enterococcus can take advantage of the excessive nutrients in the intestinal lumen to grow in large numbers [98]. After transplantation, if vancomycin-resistant enterococci are dominant, they are likely to cause VRE bacteremia, and the subsequent bacterial bloodstream infection greatly increases the posttransplant mortality rate [99].

Clostridium can produce SCFA (butyrate) to upregulate Treg cells to exert anti-inflammatory effects [100, 101]. At the same time, butyrate also promotes the recovery of IEC damage after allo-HSCT, reduces cell apoptosis, and relieves GVHD [102]. However, recent studies have shown that after the occurrence of GVHD, and butyric bacteria are associated with the development of steroid-refractory GVHD. Colonic inflammation leads to the loss of the intestinal barrier or crypts, the proliferation of IECs in the colon exposed to butyric acid produced by microorganisms is inhibited, and the recovery of the colonic mucosa is delayed. Butyrogenic bacteria may help prevent the occurrence of aGVHD, but when aGVHD occurs in the intestine, butyrogenic bacteria may impair the recovery of the intestinal mucosa, depending on the status of the colonic mucosa [103, 104]. Additionally, the increase in the abundance of cyanobacteria (Blautia) in Clostridia is related to the reduction of lethal GVHD and the improvement of the overall survival rate, while the loss of Blautia is related to the use of antibiotics to inhibit anaerobes and receiving long-term total parenteral nutrition [105]. The decrease in Lachnospiraceae and Ruminococcaceae and the increase in Enterobacteriaceae are related to the imbalance of Treg/Th17, which induces the occurrence of aGVHD [106]. Wu et al. showed that TMAO enhanced M1 macrophage polarization via NLRP3 inflammasome activation, and polarized inflammatory macrophages promoted Th1 and Th17 differentiation, which aggravated GVHD [107].

Indoles and indole derivatives, produced by symbiotic bacteria expressing tryptophan, which may enhance the integrity of the IEC epithelial barrier and reduce inflammation, are a source of normal human fecal odors. Decreased urine indoxyl sulfate (IS) levels in early post-allo-HSCT recipients (+D1 to +D10) were significantly associated with transplant-related mortality 1 year after transplantation. Thus, IS may serve as a urine marker for monitoring GVHD [84, 108]. Swimm et al. demonstrated that indoles produced by intestinal tryptophan had a protective effect against GVHD in a mouse model [109].

In healthy host immune system, diversity of intestinal microbiota is reasonable, and SIgA secreted by B cells can neutralize microbial antigens and cooperate with antimicrobial peptides (AMPs) produced by Paneth cells to prevent overgrowth of pathogens. The metabolite SCFA produced by intestinal microbiota can regulate the differentiation, recruitment, and activation of immune cells and improve the capacity to repair and protective effect of intestinal epithelial cells. Both chemotherapy and total body radiation can destroy the intestinal epithelial cells, leading to breakdown of the intestinal barrier. DAMPs released after intestinal epithelial cell death, bacterial translocation, and PAMPs can activate APC such as dendritic cells of the host, leading to the release of proinflammatory cytokines and the activation of donor T cells, thereby promoting the occurrence of GVHD. Bacteria that penetrate the intestinal wall activate and recruit neutrophils to the site of bacterial infection; then, neutrophils damage other intestinal epithelial cells by releasing ROS. B cells, Paneth cells, and mucous layer are thought to be the target cells of GVHD, and destruction of these cells exacerbates the disruption of intestinal barrier function, leading to increased mortality after allogeneic hematopoietic stem cell transplantation.

## 4. Prophylaxis and Treatment of GI GVHD after HSCT

**Figure 2:**
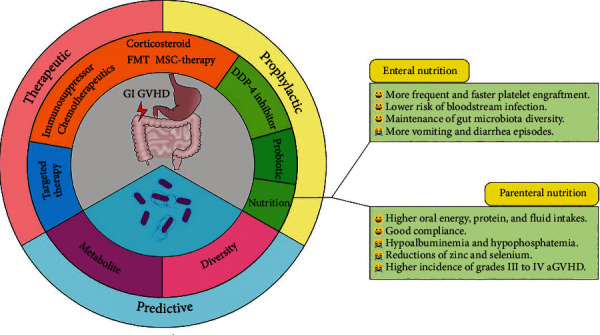
Clinical intervention used for preventing, treating and predicting GI GVHD. Clinically, DDP-4 inhibitor and probiotic are available for the prevention of GI GVHD and targeted therapy for the treatment. Compared with parenteral nutrition, enteral nutrition can prevent GI GVHD and causes fewer complications. In addition, immunosuppressor, chemotherapeutics, corticosteroid, FMT, and MSC-therapy can be used for both prevention and treatment of GI GVHD. Microbiota metabolism analyses and reduction in microbiota diversity can predict outcomes after transplantation and GI GVHD.

During allo-HSCT, GI GVHD prophylaxis is based on the rational use of various immunosuppressive agents and chemotherapeutic drugs (Figure 2). If diarrhea or abdominal pain is present, the application of glucocorticoids should be considered. Clinically, the diagnosis of GI GVHD still requires the identification of intestinal infections caused by specific pathogens (e.g., primary or reactivated cytomegalovirus infection) or drug-related side effects (e.g., MMF) [82, 110].

### 4.1. Immunosuppressor Combined with Chemotherapeutic Drugs

Tacrolimus (Tac) and CsA have been widely used for the prophylaxis of GVHD in HSCT recipients. Tacrolimus plus methotrexate and cyclosporine plus methotrexate have similar GVHD and survival rates, and they can be used in the setting of sibling or matched unrelated donor transplants. After Tac or CsA is applied, the blood concentration of the drug needs to be monitored regularly. MMF can limit the proliferation of T and B lymphocytes and suppress the immune system, and this may be an effective alternative to MTX for patients who have contraindications to methotrexate or require rapid implantation (for example, patients with Aspergillus infection) receiving MAC treatment [111].

### 4.2. Corticosteroids

With lympholytic and anti-inflammatory properties, corticosteroids are the first-line treatment for grades II to IV aGVHD or moderate to severe cGVHD, as well as newly diagnosed chronic GVHD [112]. The first-line treatment of aGVHD is methylprednisolone with an initial dose of 2 mg/kg per day. Grade II aGVHD with isolated skin or upper gastrointestinal tract manifestations can be treated with lower steroid doses, such as 1 mg/kg per day methylprednisolone or prednisone [111].

### 4.3. Targeted Therapy

For hormone-resistant graft-versus-host disease, long-term use of steroids may cause serious complications, and thus, second-line therapies are necessary. Ruxolitinib is an inhibitor of JAK1 and JAK2 and works by blocking signal transduction and the activation of transcription (STAT) pathways. In a prospective study (NCT02997280) of 75 patients with srGVHD (32 acute, 43 chronic, 41 adults, and 34 children), the overall response rate (ORR) was 75% (95% CI 57–89%) in aGVHD and 81% (95% CI 67–92%) in chronic GVHD. Overall survival was 59% (95% CI 49–74%) in the acute group and 85% (95% CI 70–93%) [113]. In a phase III trial named REACH1 (NCT02953678), 71 patients with grades II–IV SR GVHD were treated with ruxolitinib 10 mg twice daily. The Day 28 ORR was 55%, including 19 (26.8%) with complete responses. Responses were observed in the skin (61.1%), upper (45.5%) and lower (46.0%) gastrointestinal tract, and the liver (26.7%) [114]. REACH2, a phase III multicenter trial, treated 309 patients with grades II–IV SR GVHD with ruxolitinib 10 mg twice daily or the investigator's choice of therapy and confirmed significant improvements in the efficacy outcomes of SR GVHD. Ruxolitinib had a significantly higher Day 28 ORR (62% vs. 39%, *p* < 0.001) and durable overall response at Day 56 (40% vs. 22%, *p* < 0.001) [115]. Vedolizumab is a monoclonal antibody that mediates the migration of T cells to the gastrointestinal (GI) endothelium and gut-associated lymphoid tissue by blocking the *α*4*β*7 integrin. In 29 patients who received 1 to 10 doses (median 3 doses) of vedolizumab 300 mg intravenous injection as the treatment of SR GI aGVHD, the total effective rate after 6 to 10 weeks was 64%, and the total effective rate at 6 months was 54%. There were 29 SAEs, including 12 infections; 3 SAEs were thought to be related to vedolizumab, and 2 of them were infections. Among the 8 patients with confirmed gastrointestinal infections, the timing of the infection did not have a clear pattern compared with the time of starting vedolizumab treatment [116].

### 4.4. Fecal Microbiota Transplantation

FMT is a process of transferring feces from a healthy donor to a recipient whose gastrointestinal microbiota balance has been disrupted. By introducing a healthy microflora, the microbiota structure of the recipient returns to balance. FMT is a very effective method for the treatment of recurrent *Clostridioides difficile* infection (CDI). It can be administered by colonoscopy, nasogastric/duodenal tube, capsule, or enema [117]. Kakihana et al. performed FMT for the treatment of 4 patients with steroid-resistant (*n* = 3) or steroid-dependent gut aGVHD (*n* = 1), with 3 complete responses and 1 partial response [118]. In a single group pilot trial (NCT02733744), 13 patients who underwent allogeneic hematopoietic stem cell transplantation received third-party FMT capsules no later than 4 weeks after neurological engraftment. There was one treatment-related serious adverse event (abdominal pain), two patients subsequently developed acute gastrointestinal graft-versus-host disease (GI GVHD), and one patient also developed bacteremia. The median follow-up for survivors was 15 months (range, 13-20 months) [119]. In a prospective, single-center, single-arm study, after receiving FMT through the nasal duodenum to treat GI GVHD, 10 patients experienced a complete clinical response within 1 month after treatment [120]. For steroid-refractory or steroid-dependent GVHD, FMT has great therapeutic potential, but it remains to be seen whether the patient's gastrointestinal environment after transplantation can adapt to the gut microbiota of a healthy donor.

### 4.5. Cellular Therapy

Mesenchymal stromal cells (MSCs) are a type of pluripotent stem cell. When MSCs are exposed to a proinflammatory environment, they produce more anti-inflammatory cytokines, such as transforming growth factor-*β* and interleukin-10, so MSCs from the bone marrow can play an important role in regulating immune tolerance and autoimmunity and can be used to treat GVHD [121]. In a pilot study, six children were treated with decidua stromal cells (DSCs) for steroid refractory aGVHD. A complete response was observed in four children, and a partial response was observed in two children at 6 months [122]. A meta-analysis showed that infusion of MSCs prevents GvHD. The overall survival rate of the patients (95% CI, 1.02~1.33) was 17% higher than that of the control group, and the overall survival rate of the GVHD patients was positively correlated with the dose of bone marrow MSCs (*p* = 0.0214) [123]. Zhang et al. showed that mesenchymal stem cell-derived extracellular vesicles (MSC-EVs) enhanced Treg production, which is EV- and APC dose-dependent, through an APC-mediated pathway in vitro and in vivo, and MSC-EVs alleviated GVHD symptoms and increased survival in a mouse model [124]. As a soluble factor secreted by MSCs, MSC-EVs play an immunomodulatory role and may influence the bone marrow microenvironment through paracrine mechanisms [125, 126]. MSC-EVs appear to be a promising noncellular therapy for the prophylaxis and treatment of GVHD after allo-HSCT in the future.

### 4.6. Dipeptidyl Peptidase 4 Inhibitor

Dipeptidyl peptidase 4 (DPP-4 or CD26) is a T-cell costimulatory molecule expressed on T cells with a costimulatory function in activating T cells, and downregulation of CD26 prevented GVHD but preserved graft-versus-leukemia effects in a mouse model [127]. In a phase 2 nonrandomized trial using sitagliptin, which is a DDP-4 inhibitor used in combination with tacrolimus and sirolimus, aGVHD occurred in 2 of 36 patients by Day 100, and the nonrelapse mortality was zero at 1 year, which suggested that DPP-4 is a viable target for the prevention of aGVHD [128].

### 4.7. Probiotic Supplement

As the most frequently used alternative treatment, Lactobacillus spp. and Bifidobacterium spp. have been proven to be effective against a variety of diarrheal diseases, including antibiotic-associated diarrhea, infectious diarrhea or gastroenteritis, irritable bowel syndrome, ulcerative colitis, necrotizing enterocolitis, constipation, and cystitis, and can also be used to prevent *Clostridium difficile* infection [129]. Currently, the safety and feasibility of LBP in HSCT patients among high-risk children with impaired intestinal mucosal integrity have been verified by experiments. No cases of LBP bacteremia were observed in a total of 40 cases in the two trials [130, 131]. Studies in mice have shown that administration of the probiotic *Lactobacillus rhamnosus* GG reduced the incidence of GVHD after HSCT [132]. However, the effectiveness of probiotics in the prevention or treatment of GVHD still needs to be verified by more clinical studies due to the current studies having small sample sizes and low frequencies of outcomes.

### 4.8. Microbial Metabolites

Indole and indole-3-carboxaldehyde (ICA) produced by tryptophan metabolism in intestinal microbiota can limit the development of GI GVHD via type I interferon signaling but preserve antitumor responses. Swimm et al. observed that the incidence and mortality of GVHD in allo-HSCT recipient mice colonized with tryptophanase-positive strains of *Escherichia coli* or given ICA were greatly reduced. In addition, colons from ICA-treated mice at Day 21 posttransplant showed fewer pathological changes, such as crypt loss, apoptosis, and inflammation [109]. Administration of exogenous butyrate or colonization by butyrate-producing bacteria can restore butyrate levels and promote histone acetylation in IECs, which results in increased expression of antiapoptotic proteins involved in barrier integrity, thereby mitigating GVHD [133]. ICA and butyrate treatment promises to be a therapeutic option for posttransplant patients at risk for GVHD, but more studies are required to demonstrate the safety of ICA and butyrate before they can be used in clinical trials.

## 5. Conclusions

Graft-versus-host disease (GVHD) is a disease caused by T lymphocytes in allogeneic donor grafts after transplantation, which undergoes a series of “cytokine storm” stimulations initiated by the recipient, greatly enhancing their immune response to the recipient antigen and launching cytotoxic attacks on the recipient target cells. At present, increasing evidence suggests that gut commensal microbiota and its metabolites, with changes in the intestinal environment, play a positive or negative role in GI GVHD immunology. Therefore, the intestinal microecology should not be ignored when studying the interactions of immune cells with them. Research on the role of the gut microbiota in the intestinal mucosa has gradually improved. Meanwhile, FMT and probiotic supplements have been tested in clinical trials with promising results. In the future, we should devote more effort to understanding the effects of bacterial metabolites on the intestinal mucosa to develop more effective methods for the prophylaxis and treatment of GI GVHD.

## Data Availability

The original contributions presented in the study are included in the article. Further inquiries can be directed to the corresponding authors.
